# Balanced Suspension versus Pillow on Preoperative Pain for Proximal Femur Fractures: A Randomized Controlled Trial

**DOI:** 10.1155/2020/3073892

**Published:** 2020-07-21

**Authors:** Varah Yuenyongviwat, Chonthawat Jiarasrisatien, Khanin Iamthanaporn, Theerawit Hongnaparak, Boonsin Tangtrakulwanich

**Affiliations:** Department of Orthopedics, Faculty of Medicine, Prince of Songkla University, Songkhla 90110, Thailand

## Abstract

**Introduction:**

To evaluate the efficacy of a balanced suspension system, using the Thomas splint, with Pearson attachment, compared with a pillow for preoperative pain in patients with proximal femoral fractures.

**Materials and Methods:**

Sixty patients with proximal femur fractures were randomized into two groups: a balanced suspension group and a pillow group. In the first group, a balanced suspension was applied after length adjustment, to match the patient's leg and thigh. In the pillow group, a pillow was placed below the patient's leg, to position the patient's hip in a semiflexion and external rotation position. Preoperative pain severity, by using a verbal numerical rating scale (VNRS), the amount of morphine consumed, and complication were recorded.

**Results:**

There were no differences in patient characteristics between the groups. The mean VNRS for pain was not statistically different between the groups, from the start of the study up to 48 hours. The mean of morphine consumption was not different between the groups at the start of the study, on day 1, and on day 2 (*p*=0.25, 0.89, and 0.053, respectively).

**Conclusions:**

A balanced suspension did not improve patient outcome to the same level as other tractions in previous studies. Hence, other methods for reducing pain, while waiting for definite operations, should be focused on. The clinical trial is registered with TCTR20150514002.

## 1. Introduction

A fracture at the proximal femur usually occurs in the elderly because of osteoporosis [1]. Patients who have had these types of fractures have increased mortality rates of 8.4% to 36% in the first year after the fracture [2]. Complications of these fractures are pressure sore, pneumonia, and deep vein thrombosis, due to the immobility of the patient [3]. Surgical treatment with internal fixation or hip arthroplasty is the standard treatment for early ambulation [4]. However, in some conditions, patients are not able to have an operation on the same day of injury, due to underlying diseases that require preoperative management, or the operation must be delayed because of long operative schedules in some hospitals.

There are many options for preoperative intervention for patients with proximal femur fractures, such as skin traction, skeletal traction, or simple placement of a pillow under the injured limb [5–10]. However, none of these techniques have shown to have any superior effect of pain relief among other techniques [6].

The balanced suspension system is familiar to practitioners in clinical practice. This technique is an immobilization technique, which allows the proximal and distal parts of the fractured limb to float above the bed [11]. A balanced suspension supports the extremity and allows movement of the injured limb, along with the patient's body, when the patient moves. Therefore, patients should, theoretically, have less pain compared with other immobilization methods.

Therefore, the primary outcome of this study was the evaluation on the efficacy of balanced suspension, using the Thomas splint with Pearson attachment, on preoperative pain compared with a pillow while patients are waiting for an operation.

## 2. Materials and Methods

The inclusion criteria were patients aged 60 or above, with closed femoral neck fractures or intertrochanteric fractures. The excluded patients were those with multiple fractures, pathological fracture from the neoplasm and abnormal neurological system, patients allergic to the medications in this study, and patients who had cognitive impairment or could not communicate. Finally, all patients provided written informed consent.

This trial was approved by the Ethics Committee and Institutional Review Board. The procedures in this study were in accordance with the Declaration of Helsinki on ethical principles for medical research involving human subjects.

This was a prospective randomized controlled trial. Blocks of four, using a computer-generated random number, were used to randomize the patients into two groups. Allocation concealment was performed by opaque, sealed envelopes. The patients were randomized at the time of admission in the ward. Each group had 30 patients. In the first group, the fracture was immobilized by using a balanced suspension (a Thomas splint with a Pearson attachment), for the fractured limb. In other group, a polyester pillow (size: 45 × 13 × 62 centimeters, weight: 1200 grams) was placed under the fractured limb.

**Figure 1:**
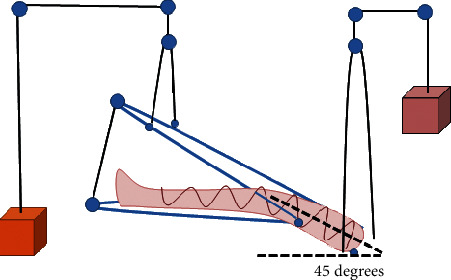
Setup of the balanced suspension, using the Thomas splint with the Pearson attachment.

Balanced suspension was applied after length adjustment to match the patient's leg and thigh. The proximal part was placed for support of the thigh, whilst the distal part supported the leg. The joint position of the splint was placed at the knee joint. The proximal and distal parts of the splint were hung separately with metal weights. Each part was hung with 1/12 of the patient's body weight (Figure 1). After applying the weight, the patient's ischial tuberosity floated above the bed at 1-2 centimeters. In this study, the patient's hip was placed at a 45 degree angle of flexion, and the leg was floating parallel to the bed. In the pillow group, a pillow was placed below the patient's leg, so as to position the patient's hip in a semiflexion and external rotation position.

All patients were continuously on either a balanced suspension or a pillow, from admission until hip surgery was performed. All patients received a 500 mg paracetamol tablet every six hours for pain control. Intravenous morphine (2 mg) or fentanyl (20 *μ*g) was used as rescue medication, if the level of pain via the verbal numeric rating scale (VNRS, 0 (no pain) to 10 (worst imaginable pain)) was 4 or higher or if the patient required additional analgesics. All patients had an inserted urinary catheter, which was connected to a drainage bag in the operative room. The catheters were removed on the day after the operation.

Pain scores were recorded using the verbal numeric rating scale (VNRS), which is the standard scale for pain evaluation. Scores were validated in another study [12]. The pain scores were recorded before and immediately after positioning the patient in either the balanced suspension or by placement of the pillow and, then, subsequently at 15 mins and every 8 hr. The pain score and morphine consumption were recorded by a nurse. Both the pain score and morphine consumption from the start of the study to 48 hours were analyzed.

R version 3.1.0 software (R Foundation for statistical computing, Vienna, Austria) was used for the data analyses. Patient demographic data, in terms of age, were compared by Student's t-test. The gender, injured side, American Society of Anesthesiologists (ASA) classification, and type of fracture were analyzed by the Chi-squared test. Body weight and time to surgery were evaluated using the Wilcoxon rank-sum test. The pain score and morphine consumption were compared by Student's t-test. Complications, such as pressure ulcer, urinary tract infection, pneumonia, deep vein thrombosis, and delirium, were analyzed using Fisher's exact test. The primary outcome was analyzed on the intention-to-treat.

The sample size was estimated from a previous study [7]. Twenty-seven patients, per study group, were required to detect 2 score differences in the VNRS for pain between the groups, with a significance level set at 0.05 and power set at 0.9.

## 3. Results

**Figure 2:**
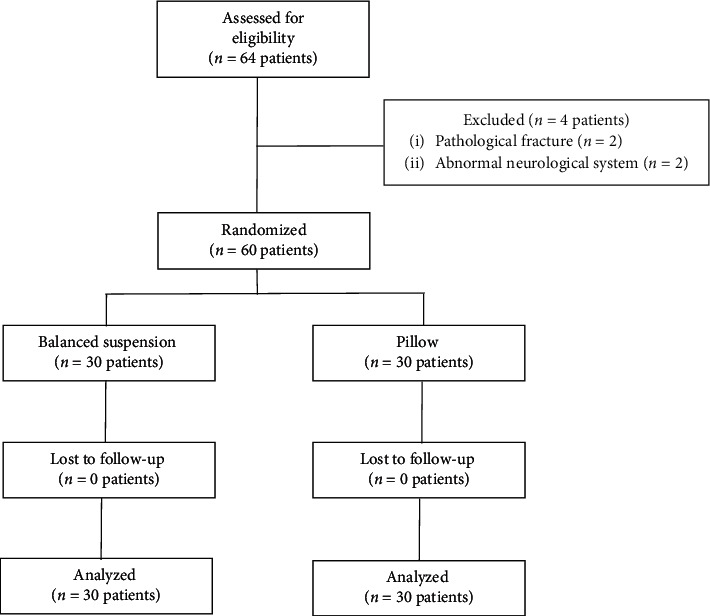
Flow study diagram.

Sixty-four patients were recruited in this study, with four patients being excluded due to exclusion criteria. Finally, 60 patients were included into the study and were analyzed (Figure 2).

Differences in the demographic data, for example, age, gender, ASA classification, weight, the type of fracture, and the side of injury, were not statistically significant between the groups (Table 1).

**Figure 3:**
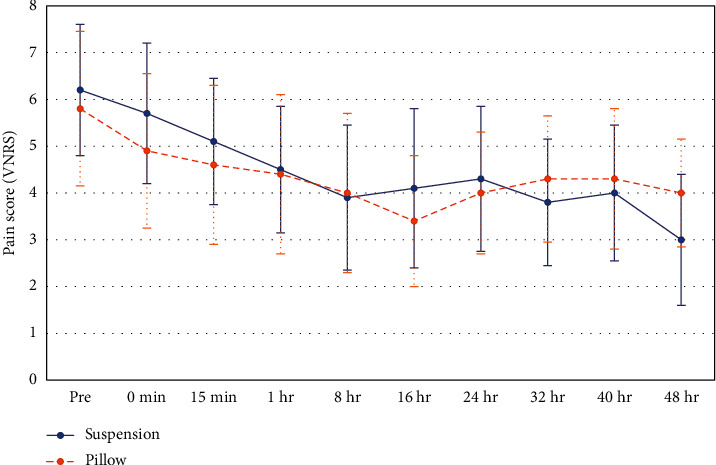
Means of the verbal numeric rating scale (VNRS) for pain by time.

The mean VNRS for pain was not different between the groups at the start of the study, immediately after the applied intervention, or at 15 mins, 1 hr, 8 hrs, 16 hrs, 24 hrs, 32 hrs, and 40 hrs or until 48 hours had lapsed (*p* = 0.58, 0.35, 0.55, 0.9, 0.9, 0.35, 0.78, 0.51, 0.73, and 0.23, respectively) (Figure 3).

The means of morphine consumption were not different between the groups at the start of the study (pillow group: 0.3 mg (SD, 0.6) versus the balanced suspension group: 0.6 mg (SD, 1.3), *p*=0.25), on day 1 (pillow group: 1.8 mg (SD, 2.3) versus the balanced suspension group: 1.9 mg (SD, 3.1), *p*=0.89), and on day 2 (pillow group: 0.9 mg (SD, 1.2) versus the balanced suspension group: 2 mg (SD, 2.5), *p*=0.053).

The incidence of complications was higher in the pillow group (1 pulmonary embolism, 2 urinary tract infections, 1 pneumonia, 1 delirium, and 1 atelectasis) compared with the balanced suspension group (1 delirium). The incidence of complications was statistically different between the groups (*p*=0.04).

## 4. Discussion

Proximal femur fractures are painful and can cause complications while the patient is waiting for definitive surgery. There are many interventions to either immobilize or adjust a limb, so as to reduce pain in patients with skeletal fractures, for example, skin traction and a pillow, but none of them show superiority to the others [5–7]. Balanced suspension is an intervention that can reduce the gravitational load on the extremity. It was reported for use in postoperative treatment of hip fractures, as well as arthroplasties of patients [11]. This method is a preoperative option for patients who are not suitable for an early operation, due to unstable medical conditions or long operating room waiting lists in some hospitals. Therefore, the authors evaluated the efficacy of the balanced suspension technique, using the Thomas splint with the Pearson attachment, on preoperative pain compared with a pillow.

Preoperative pain was not different between the balanced suspension group and the pillow group in this study. This was similar with previous randomized control studies. Anderson et al. reported no differences in terms of pain levels in patients with proximal femur fractures, as compared with skin traction and nursed free in bed [13]. Saygi et al. studied the efficacy of skin traction using 2 kg of weights, skin traction without weights, and pillow placement under the affected limb [14]. They found that patients treated with skin traction using 2 kg of weights compared with patients in a pillow group were not statistically different in terms of pain. However, patients who had skin traction applied without weights had a statistically significant reduction in pain, which they stated was possibly due to the placebo effect.

In this study, morphine consumption was not different between the balanced suspension group and the pillow group, over a period of 0 to 48 hrs. A randomized study by Yip et al. reported that the analgesic requirements were not different in patients with skin traction or a pillow below the limb, from day 0 to day 7 [15].

Complications in the balanced suspension group were statistically lower than those in the pillow group in this study. These results were contrary to a previous study by Anderson et al. who reported the same rates of complications in skin traction and nursed free in bed [13]. However, the complication rate in our study was a total complication rate, which could not be compared separately for each complication, due to the limit of the sample size. Our results might have happened by chance, or it might be possibly because the patients in the balanced suspension group could move their bodies or limbs more than those in the pillow group. Balanced suspension can, theoretically, support their limbs so as to move along with their body. Further studies with a larger number of patients would be able to evaluate complications in more detail.

This study had a number of limitations. First, our study could not blind the patients from the intervention. Second, the assessor was also not blinded to the groups of patients, due to the need to record data frequently, while the patient was on the bed with the intervention. However, the assessor was not involved with the study. Third, post hoc analysis found that this study was underpowered, due to the lower-than-expected differences in pain scores. The number of participants to detect differences in pain at 48 hours, after fracture, with a significance level set at 0.05, and power set at 0.8 should be 104 patients per group. Although there were some limitations, we believe that our study was a pilot study that provides useful information, suggesting further study in this topic.

## 5. Conclusions

This study found no differences in either pain scores or morphine consumption between balanced suspension and pillow groups, from the start of the study up to 48 hours. The balanced suspension did not improve patient outcome to the same level as other tractions used in previous studies. Therefore, other methods for reducing pain, while waiting for a definite operation, should be focused on.

## Figures and Tables

**Table 1:** Demographic data.

Demographic data	Balanced suspension *n* = 30	Pillow *n* = 30	*p* value
Age (years)	79.3 (7.9)^*∗*^	81.4 (7.9)^*∗*^	0.30
Gender (male:female)	7 : 23	9 : 21	0.77
Weight (kg)	50 (45,55.8)^*∗∗*^	50.5 (42,57.8)^*∗∗*^	0.85
Side (right:left)	16 : 14	16 : 14	1.00
ASA classification (II : III)	21 : 9	19 : 11	0.58
Type of fracture			0.12
(i) Intertrochanteric fracture	13	20	
(Evan's stable/unstable)	(6/7)	(12/8)	
(ii) Femoral neck fracture	17	10	
(Garden's III/IV)	(7/10)	(4/6)	
Time to surgery (hours)	69 (44.5,93.2)^*∗∗*^	59 (24.8,96.2)^*∗∗*^	0.40
Treatment			0.12
(i) Internal fixation	13	20	
(ii) Hip replacement	17	10	

^*∗*^Mean (SD); ^*∗∗*^Median (IQR).

## Data Availability

The datasets generated during and/or analyzed during the current study are available from the corresponding author upon reasonable request.
